# Novel efficacious microRNA-30c analogs reduce apolipoprotein B secretion in human hepatoma and primary hepatocyte cells

**DOI:** 10.1016/j.jbc.2022.101813

**Published:** 2022-03-10

**Authors:** Pradeep Kumar Yadav, Phensinee Haruehanroengra, Sara Irani, Ting Wang, Abulaish Ansari, Jia Sheng, M. Mahmood Hussain

**Affiliations:** 1 Department of Foundations of Medicine, NYU Long Island School of Medicine, Mineola, New York, USA; 2 Department of Chemistry, The RNA Institute, University at Albany, SUNY, Albany, New York, USA; 3 Department of Cell Biology, SUNY Downstate Medical Center, Brooklyn, New York, USA; 4 Research Department, VA New York Harbor Healthcare System, Brooklyn, New York, USA

**Keywords:** lipids, lipoproteins, microsomal triglyceride transfer protein, modified microRNAs, GalNAc, liver, apoA1, apolipoprotein A1, apoB, apolipoprotein B, cDNA, complementary DNA, DMEM, Dulbecco's modified Eagle's medium, FBS, fetal bovine serum, GalNAcαProN3, 3-azidopropyl 2-acetamido-2-deoxy-α-d-galactopyranoside, miR, microRNA, miR-30c, microRNA-30c, MTP, microsomal triglyceride transfer protein, 2′-OMe, 2′-*O*-methyl

## Abstract

High plasma lipid levels have been demonstrated to increase cardiovascular disease risk. Despite advances in treatments to decrease plasma lipids, additional therapeutics are still needed because many people are intolerant or nonresponsive to these therapies. We previously showed that increasing cellular levels of microRNA-30c (miR-30c) using viral vectors or liposomes reduces plasma lipids and atherosclerosis. In this study, we aimed to synthesize potent miR-30c analogs that can be delivered to hepatoma cells without the aid of viral vectors and lipid emulsions. We hypothesized that modification of the passenger strand of miR-30c would increase the stability of miR-30c and augment its delivery to liver cells. Here, we report the successful synthesis of a series of miR-30c analogs by using different chemically modified nucleosides. In these analogs, we left the active sense strand untouched so that its biological activity remained unaltered, and we modified the passenger strand of miR-30c to enhance the stability and uptake of miR-30c by hepatoma cells through phosphorothiorate linkages and the addition of GalNAc. We show that these analogs significantly reduced apolipoprotein B secretion in Huh-7 human hepatoma cells and human primary hepatocytes without affecting apolipoprotein A1 secretion and cellular lipid levels. Our results provide a proof of concept that the passenger strand of miR-30c can be modified to increase its stability and delivery to cells while retaining the potency of the sense strand. We anticipate these miR-30c analogs will be useful in the development of more efficacious analogs for the treatment of hyperlipidemias and cardiovascular diseases.

Atherosclerosis, hardening of the arteries after lipid deposition, is the leading cause of morbidity and mortality in the United States and worldwide. High plasma cholesterol levels are a major risk factor for atherosclerosis. Cholesterol in the circulation is carried primarily by apolipoprotein B (apoB) containing lipoproteins. Remarkable advances have been made in lowering plasma cholesterol and reducing death by 30 to 40% through treatments with statins and proprotein convertase subtilis/kexin type 9 inhibitors (1, 2, 3, 4). Despite the availability of these drugs, an unmet need for new lipid-lowering therapies remains, because some patients do not achieve the desirable cholesterol lowering with statins (5); a substantial proportion of patients experience unmanageable adverse effects (6, 7); and statins and proprotein convertase subtilis/kexin type 9 antibodies are not useful in treating patients with homozygous familial hypercholesterolemia and low-density lipoprotein receptor null mutations (8, 9). Therefore, a prevailing need exists to identify safer methods of lowering plasma lipids that can be used independently of or in combination with statins and other available drugs.

Statins, inhibitors of hydroxyl-methyl-glutaryl-coenzyme A reductase, lower plasma lipids by increasing the hepatic expression of low-density lipoprotein receptors and decreasing cholesterol synthesis. A complementary approach involves inhibiting the assembly and secretion of lipoproteins to limit their entry into the circulation. Lipoprotein assembly requires two proteins: the structural protein apoB and the chaperone microsomal triglyceride transfer protein (MTP). MTP physically interacts with and transfers lipids in the endoplasmic reticulum to nascent apoB and assists in the formation and maturation of lipoprotein particles for secretion (10, 11). MTP has long been a drug target for lowering plasma lipids, as its biochemical activity of transferring lipids can be easily measured in laboratory settings. Several pharmaceutical companies have developed drugs that potently inhibit MTP activity and lower plasma lipids (12, 13). However, these drugs increase hepatic lipids and plasma transaminases (14, 15, 16). One MTP inhibitor, lomitapide, has been approved for the treatment of homozygous familial hypercholesterolemia on a restricted protocol and carries label warnings for hepatic steatosis (17, 18). Hence, a need remains for agents that can reduce levels of MTP and plasma lipids without causing steatosis.

MicroRNAs (miRs) are endogenous gene products ∼22 nucleotides in length that regulate gene expression at the post-transcriptional level. They interact with the 3′-untranslated regions of target mRNAs and decrease protein synthesis by enhancing mRNA degradation and/or interfering with translation (19, 20). Currently, several miR-based drugs are in clinical trials for the treatment of atherosclerosis, heart failure, diabetes, and hepatitis C viral infection and are expected to be possible treatments in the future (21, 22). A major hurdle in the development of miR therapeutics involving chemical modifications is the loss of mRNA silencing. Consequently, several methods of delivery have been devised, including viral vectors and neutral lipid emulsions (21).

MiR-30c is a small (23 nucleotides), double-stranded, and noncoding RNA. The 5′-physiologically active sense strand interacts with different mRNAs and subsequently modulates the synthesis of various proteins (23, 24). MiR-30c is derived from the products of two genes (*MIR30C1* and *MIR30C2*) in humans and mice (23). The primary transcripts of these genes, pri-miR-30c-1 and pri-miR-30c-2, show 56.7% and 58.5% similarity, respectively, between humans and mice. These transcripts are processed in the nucleus and exported to the cytoplasm as pre-miR-30c-1 and pre-miR-30c-2. Pre-miR-30c-1 is highly similar between humans and mice (98.87% similarity); in contrast, pre-miR-30c-2 is 82.1% similar between both species. These pre-miRs are further processed in the cytoplasm, thus resulting in the production of mature miR-30c with identical 5′-strands (miR-30c-5p) that are conserved in humans and mice. The 3′-strands (miR-30c-3p) derived from the two genes are slightly different but are conserved in humans and mice.

We have reported that overexpression of miR-30c significantly reduces MTP activity, whereas overexpression of its corresponding anti-miR elevates MTP activity in Huh-7 human hepatoma cells and human primary hepatocytes (25, 26, 27). Furthermore, miR-30c significantly reduces apoB secretion, whereas anti-miR-30c increases apoB secretion without affecting apolipoprotein A1 (apoA1) secretion in these cells. Mechanistic studies revealed that miR-30c decreases MTP activity by interacting with and degrading RNA at the post-transcriptional level (25, 28, 29). MiR-30c interacts with MTP mRNA involving both the seed and supplementary sites (26). Thus, miR-30c interacts with MTP mRNA and subsequently causes mRNA degradation and reduces MTP activity, thereby inhibiting apoB secretion in liver cells.

To investigate whether miR-30c regulates MTP activity and plasma lipids *in vivo*, we intravenously transduced male C57BL/6 mice with lentiviruses for the expression of control, miR-30c, or anti-miR-30c and then fed them a Western diet (25). MiR-30c decreased hepatic MTP expression, plasma cholesterol, and hepatic lipoprotein production. Despite reductions in plasma cholesterol, we did not detect increases in hepatic lipids and plasma transaminases in miR-30c-expressing mice. Mechanistic studies indicated that miR-30c might prevent hepatic steatosis by additionally targeting genes involved in lipid synthesis, such as *LPGAT1*. Because viral therapy is formidable, we intravenously injected miR-30c analogs complexed with lipid emulsions (28). These emulsions enabled delivery of miR-30c to the liver and diminished diet-induced hypercholesterolemia in the mice. Furthermore, we found that miR-30c mimic significantly reduces hypercholesterolemia and atherosclerosis in *Apoe*^*−/−*^ mice (28). Subsequent studies demonstrated that miR-30c also decreases plasma cholesterol in diabetic *ob/ob* and *db/db* mice and in Western-diet fed *Ldlr*^*−/−*^ mice but has no effect on plasma triglycerides, glucose, and transaminases (29). These studies have indicated that the hepatic expression of miR-30c decreases plasma cholesterol, hepatic lipid synthesis, and atherosclerosis without causing steatosis seen with MTP inhibitors. Therefore, miR-30c might be a superior therapeutic agent to treat hyperlipidemia and atherosclerosis in patients with statin intolerance and homozygous familial hypercholesterolemia.

In the studies summarized previously (25, 28, 29), miR-30c was injected intravenously as lentiviruses or complexed with lipid emulsions. Because these approaches can be expensive and difficult for therapeutic interventions, we aimed to synthesize miR-30c analogs deliverable to cells without using lipid emulsions or viral vectors. We show that the miR-30c-3p passenger strand can be modified and duplexed with native miR-30c-5p to augment delivery to hepatoma cells and to reduce apoB secretion without affecting apoA1 secretion.

## Results

### Modified miR-30c duplexes have increased thermal stability and are physiologically active

**Figure 1:**
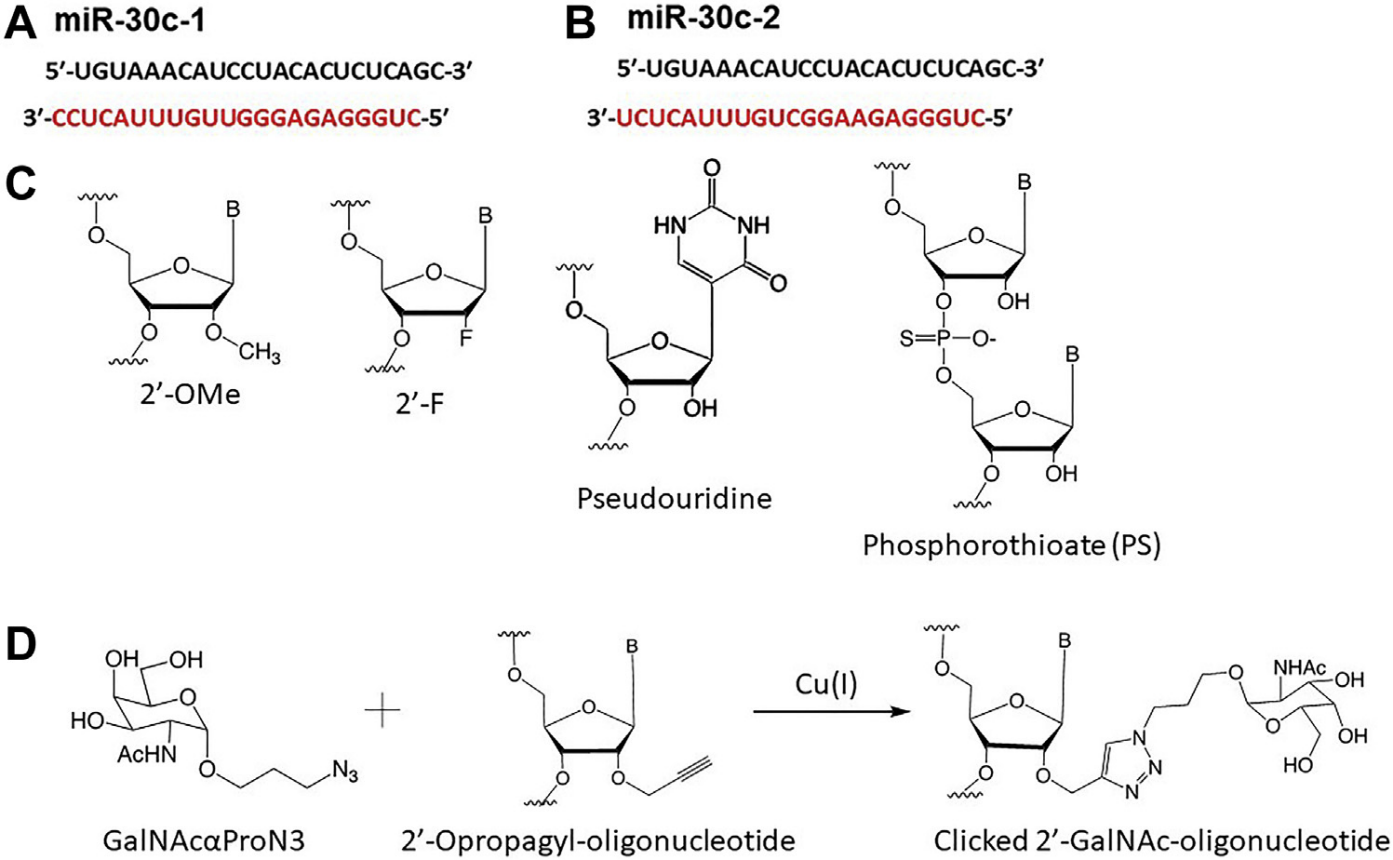
**MiR-30c sequences and several commonly used RNA modifications.***A* and *B*, the two double-stranded mature miR-30c sequences derived from two independent genes are shown. The two passenger strands, miR-30c-1 and miR-30c-2, are shown in *red*. The common miR-30c-5p strand is shown in *black*. *C*, different chemically modified nucleotides used in the synthesis of modified miR-30c passenger strands. *D*, synthesis of GalNAc cytidine. miR-30c, microRNA-30c.

**Figure 2:**
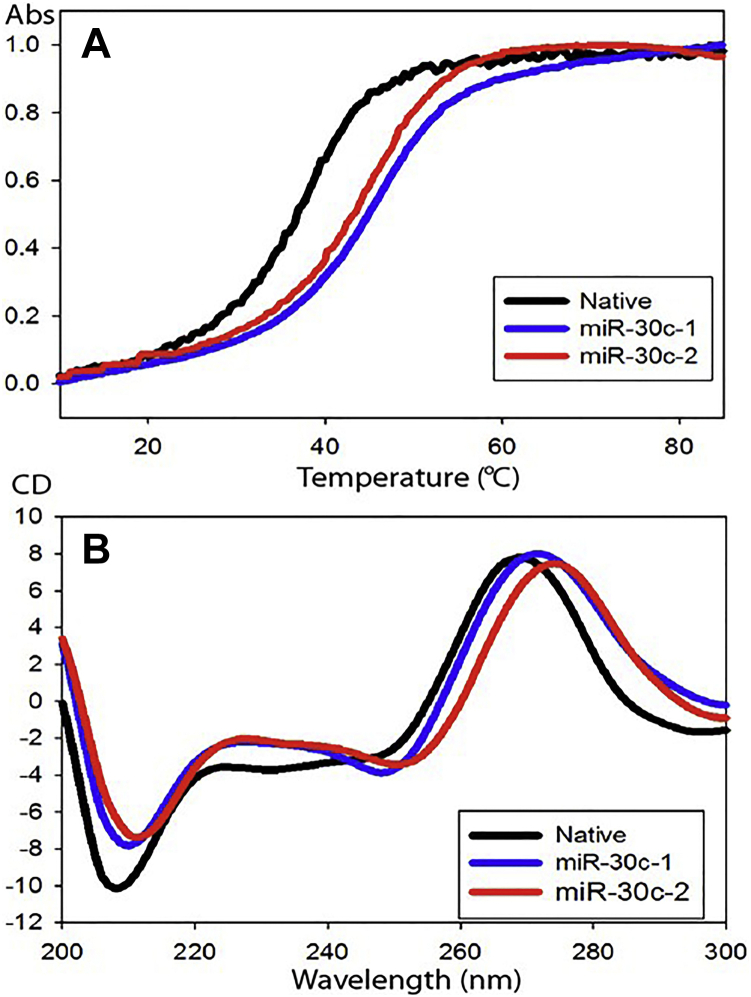
**Thermal stability and CD spectra of modified miR-30c duplexes.***A*, melting temperature and (*B*) CD spectra of native miR-30c, and 2′-OMe-modified miR-30c-1-3p and miR-30c-2-3p duplexed with native miR-30c-5p strand. miR-30c, microRNA-30c.

RNA interference–based therapy is becoming a feasible and attractive approach for treating life-threatening diseases that are not easily treatable through conventional small drug molecules. The success of siRNA-based therapy has been due to improvements in the stability, potency, specificity, delivery, and safety of modified siRNAs (30, 31, 32). Building on prior advances made in siRNA delivery technology, we synthesized novel analogs of miR-30c to assess whether these approaches might be extended to miRs. Strategically, we decided to modify only the antisense strand (passenger strands, miR-30c-1-3p and miR-30c-2-3p) while leaving the active sense strand (guide strand, miR-30c-5p) untouched to avoid hindering its ability to interact with the RNA-induced silencing complex and recognition of target mRNAs. As shown in Figure 1, *A* and *B*, two versions of double-stranded mature miR-30c sequences (miR-30c-1 and miR-30c-2) are derived from the products of two independent genes, *MIRC1* and *MIRC2*, and contain an identical guide strand (sense strand, miR-30c-5p, 5′-UGU AAACAUCCUACACUCUCAGC-3′) but slightly different passenger strands (antisense strands, *red*, miR-30c-1-3p, 5′-CUGGGAGAGGGUUGUUUACUCC-3′ and miR-30c-2-3p, *black*, 5′-CUGGGAGAAGGCUGUUUACUCU-3′). First, we synthesized modified miR-30c-1-3p and miR-30c-2-3p passenger strands by using all 2′-*O*-methyl (2′-OMe) nucleosides (Fig. 1*C*), then annealed these antisense strands with a sense unmodified or native miR-30c-5p strand to study the biophysical properties of the annealed duplexes. For a control, we annealed unmodified miR-30c-1-3p with native miR-30c-5p. Duplexes of miR-30c-5p with native miR-30c-3p, modified miR-30c-1-3p, or modified miR-30c-2-3p showed similar denaturation curves (Fig. 2*A*). However, the overall thermal stability of duplexes with modified miR-30c-3p strands increased by ∼7 °C, thus indicating enhanced stability of the duplexes. The CD spectra showed similar conformations for both native and modified duplexes. These duplexes had a strong positive peak in the range of 260 to 270 nm (Fig. 2*B*). Thus, the annealing of miR-30c antisense strand containing modified base pairs does not affect its interaction with the miR-30c sense strand in duplex formation and the double-helix conformation.

**Figure 3:**
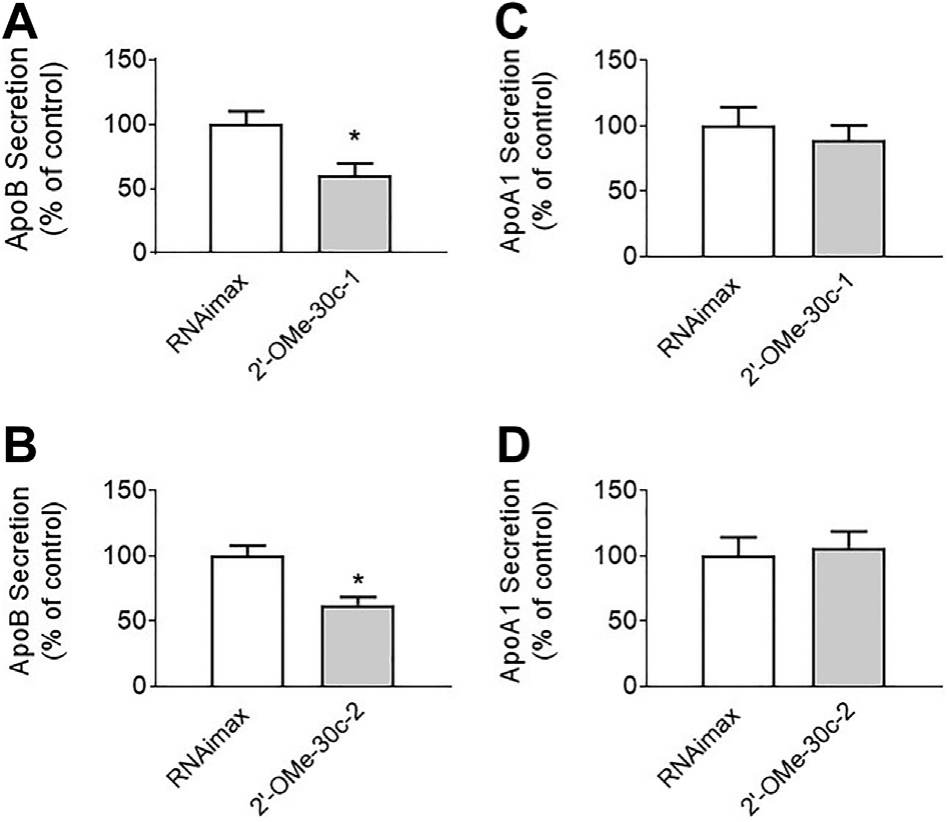
**Activity of 2′-OMe modified miR-30c-1 and miR-30c-2 in Huh-7 human hepatoma cells.** Huh-7 cells were reverse transfected with 100 nM of 2′-OMe-modified miR-30c analogs complexed with Lipofectamine RNAiMax transfection reagent at a ratio of 3:1. For the control, cells were treated only with Lipofectamine RNAiMax transfection reagent (no mimics). Forty-eight hours after transfection with 2′-OMe-30c-1 and 2′-OMe-30c-2 analogs, media were collected and used to measure apoB and apoA1 concentrations. Data are representative of three independent experiments. Significance was determined at *p* < 0.05 (∗) with two-tailed *t* test, and error bars represent mean ± SD (*A*–*D*). apoA1, apolipoprotein A1; apoB, apolipoprotein B; miR-30c, microRNA-30c.

These data indicated that the synthesis of miR-30c-3p with modified nucleotides does not affect either the ability of these modified passenger strands to bind the sense strand or the stability of the dsRNA. Therefore, we evaluated the efficacy of theses miRs in reducing apoB secretion in Huh-7 human hepatoma cells (Fig. 3) by adding them to the media and analyzing their effects on apoB secretion. The duplexes had no effect on apoB secretion when added to cells without transfection reagent. These negative results might have been because the duplexes failed to be delivered to the cells or were not physiologically active. To determine the reasons for their inactivity, we introduced native miR-30c-5p complexed with 2′-OMe modified miR-30c-1-3p (2′-OMe-30C-1) or miR-30c-2-3p (2′-OMe-30c-2) analogs into cells with Lipofectamine RNAiMax transfection reagent (Invitrogen). Both modified complexes significantly reduced apoB secretion (Fig. 3, *A* and *B*) without affecting apoA1 secretion (Fig. 3, *C* and *D*), thus indicating that the modified miRs were physiologically active when introduced as lipid complexes. The inability of these complexes to reduce apoB secretion in the absence of lipid-mediated delivery was probably because the RNAs were unable to enter the cells. These data provided crucial preliminary evidence that miR-30c-3p modifications are tolerable if delivered to cells and suggested the feasibility of synthesis of more potent miR-30c analogs by modifying the antisense strands of miR-30c.

**Figure 4:**
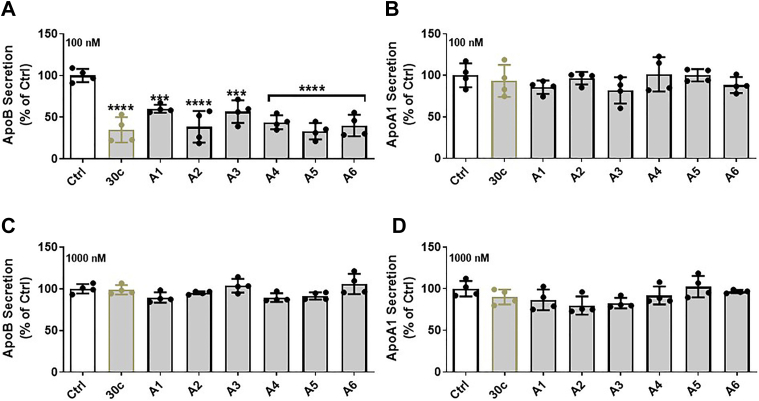
**Effects of different miR-30c series A analogs on apoB secretion in Huh-7 human hepatoma cells.***A*, apoB secretion in Huh-7 cells transfected with control (Ctrl) and different miR-30c analogs (100 nM), as described in Table 1, with Lipofectamine RNAiMax transfection reagent. MiR-30c (30c) was used as a positive control. *B*, apoA1 secretion in Huh-7 cells transfected with control (Ctrl) and different miR-30c analogs (100 nM) with Lipofectamine RNAiMax transfection reagent. *C*, apoB secretion in Huh-7 cells transfected with Ctrl and different miR-30c analogs (1000 nM) without Lipofectamine RNAiMax transfection reagent. *D*, apoA1 secretion in Huh-7 cells transfected with Ctrl and different miR-30c analogs without Lipofectamine RNAiMax transfection reagent. The apoB and apoA1 concentrations were measured by ELISA in the collected media. Data are representative of four independent experiments. Significance was determined at *p* < 0.0001 (∗∗∗∗) and *p* < 0.001 (∗∗∗) with one-way ANOVA, and error bars represent mean ± SD (*A*–*D*). apoA1, apolipoprotein A1; apoB, apolipoprotein B; miR-30c, microRNA-30c.

Next, we introduced pseudouridine (ψ) (Fig. 1*C*) in place of the natural uracil and synthesized six different series A (A1–A6) strands (Table 1). Pseudouridine stabilizes the tertiary structure of tRNA, and the synthetic replacement of all uracils with pseudouridine renders mRNA nonimmunogenic and increases its stability (33, 34). All analogs were transfected into Huh-7 cells using Lipofectamine RNAiMax transfection reagent (Fig. 4*A*). For the positive control, we used commercial native miR-30c. For the negative control, we used an unrelated commercially available miR (Ctrl). Compared with the Ctrl, miR-30c and all A-series analogs significantly decreased apoB secretion (Fig. 4*A*), but apoA1 secretion was unaffected (Fig. 4*B*). These studies indicated that when delivered to cells, these new synthetic analogs were able to diminish apoB secretion similarly to miR-30c, as compared with Ctrl miR. Next, we asked whether any of these analogs might affect apoB secretion without lipid emulsions. For this purpose, we provided higher concentrations of analogs to cells without Lipofectamine RNAiMax transfection reagent. These analogs and native miR-30c had no effect on apoB and apoA1 secretion (Fig. 4, *C* and *D*). These studies indicated that these analogs were unable to penetrate cells on their own to reduce apoB secretion; however, they were able to reduce apoB secretion when introduced into cells with Lipofectamine RNAiMax transfection reagent.

**Table 1:** MiR-30c analogs and sequences

Compound	Short form	Strand	Sequence (5′ to 3′)
Ctrl	Ctrl	Sense	UCACAACCUCCUAGAAAGAGUAGA
miR30c	30c	miR-30c-5p	UGUAAACAUCCUACACUCUCAGC
miR30c-A1	A1	miR-30c-1-3p	CugggagaggguuguuuacucC
miR30c-A2	A2	miR-30c-1-3p	CψgggagagggψψgψψψacψcC
miR30c-A3	A3	miR-30c-1-3p	CψgggAgAgggψψgψψψACψCC
miR30c-A4	A4	miR-30c-2-3p	CugggagaaggcuguuuacucU
miR30c-A5	A5	miR-30c-2-3p	cψgggagaaggcψgψψψacψcU
miR30c-A6	A6	miR-30c-2-3p	CψgggAgAAggCψgψψψACψCU

Underlined upper case letters, native nucleosides; lower case letters, 2′-OMe ribosugar modifications; **ψ**, pseudouridine.

### MiR-30c-3p analogs modified with GalNAc reduce apoB secretion without the use of lipid emulsions

**Figure 5:**
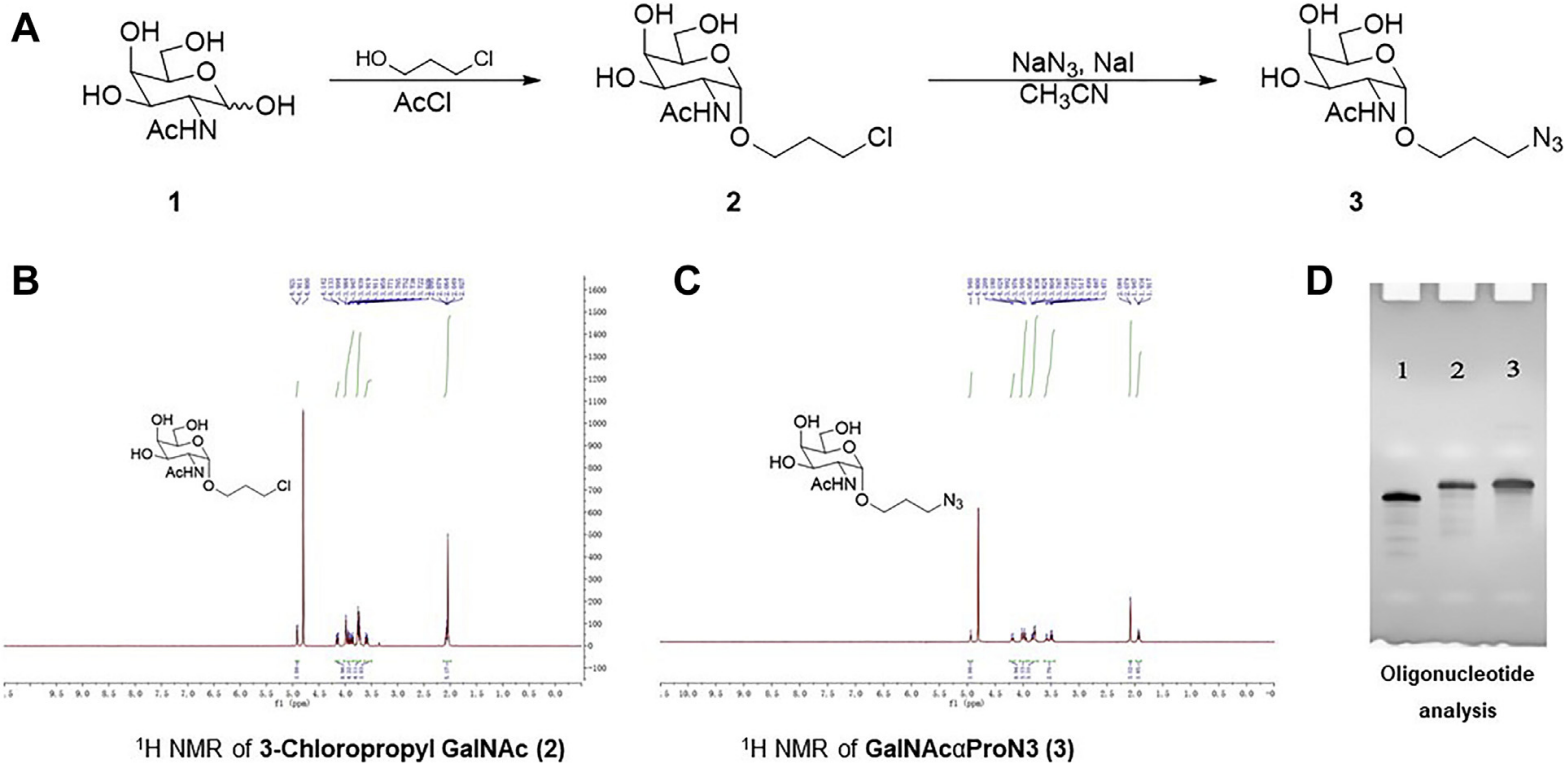
**Synthesis of 3-chloropropyl GalNAc 2 and GalNAcαProN3 3.***A*, 3-Chloropropyl GalNAc 2 and GalNAcαProN3 3 were synthesized as described in the Experimental procedures section. *B* and *C*, ^1^H NMR spectra of 3-chloropropyl GalNAc 2 and GalNAcαProN3 3 are shown. *D*, analytical 15% polyacrylamide 8 M urea gel electrophoresis of antisense-oligonucleotide (ASO) in lane 1, postclicked GalNAc-ASO in lane 2, and reference GalNAc-ASO in lane 3. GalNAcαProN3, 3-azidopropyl 2-acetamido-2-deoxy-α-d-galactopyranoside.

**Figure 6:**
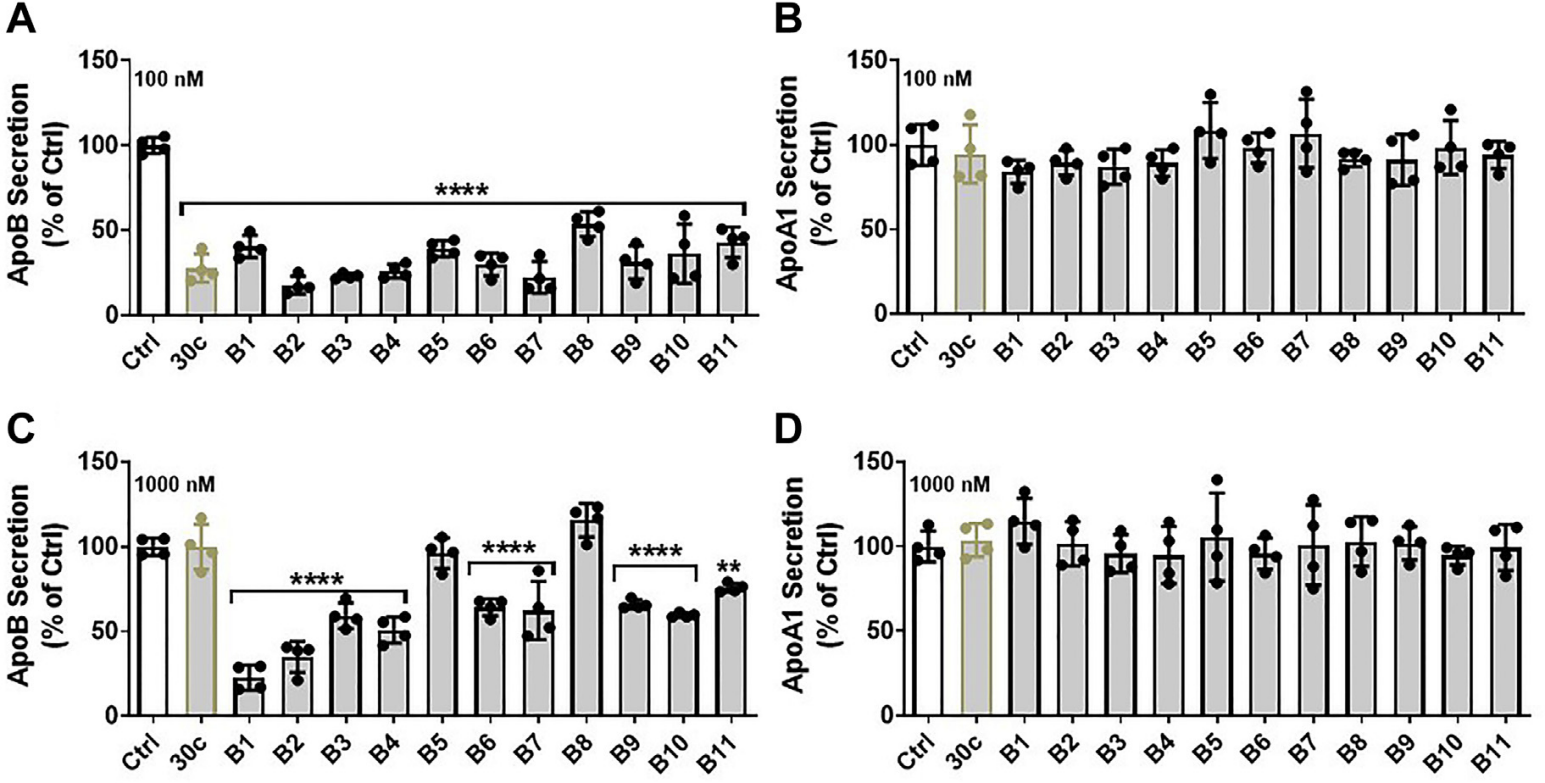
**Effects of various GalNAc-modified miR-30c analogs on apoB secretion in Huh-7 human hepatoma cells.***A*, apoB secretion and (*B*) apoA1 secretion in Huh-7 cells transfected with control (Ctrl) and different miR-30c series B analogs (Table 2) with Lipofectamine RNAiMax transfection reagent. MiR-30c (30c) was used as a positive control. *C*, apoB and (*D*) apoA1 secretion in Huh-7 cells transfected with Ctrl and different miR-30c analogs without Lipofectamine RNAiMax transfection reagent. The apoB and apoA1 concentrations in the media were measured with ELISA. Data are representative of four independent experiments. Significance was determined at *p* < 0.0001 (∗∗∗∗) and *p* < 0.001 (∗∗∗) with one-way ANOVA, and error bars represent mean ± SD (*A*–*D*). apoA1, apolipoprotein A1; apoB, apolipoprotein B; miR-30c, microRNA-30c.

In recent years, the asialoglycoprotein receptor ligand GalNAc has been used to deliver antisense and siRNA oligonucleotides to liver cells (35, 36, 37, 38, 39, 40, 41). Therefore, we introduced one GalNAc-modified nucleotide at both ends as well as more than one GalNAc-modified residue in the antisense strands. First, we synthesized 3-chloropropyl GalNAc (Figs. 5*A* and 2) and 3-azidopropyl 2-acetamido-2-deoxy-α-d-galactopyranoside (GalNAcαProN3; Figs. 5*A* and 3). Their correct synthesis was confirmed by ^1^H NMR spectra (Fig. 5, *B* and *C*) and 15% polyacrylamide analytical 8 M urea gel electrophoresis (Fig. 5*D*). Second, the GalNAcProN3 was attached to 2’-(O-propargyl)-cytosine through click chemistry (42, 43). Third, GalNAc-cytidine (pC) was used during the synthesis of miR-30c-3p strands (Table 2). During these syntheses, we also incorporated 2′-deoxy-2′-fluoro ribosugar-modified nucleosides to increase the biological stability (44) of these RNA strands (Table 2). Fourth, these analogs were transfected into cells with Lipofectamine RNAiMax transfection reagent. All analogs potently inhibited apoB secretion in Huh-7 cells (Fig. 6*A*) and therefore were biologically active. apoA1 secretion was unaffected by these analogs (Fig. 6*B*). Fifth, we evaluated the efficacy of these series B analogs in reducing apoB secretion when provided to cells without Lipofectamine RNAiMax transfection reagent. Similarly to miR-30c, compounds B5 and B8 had no effect on apoB secretion with respect to the Ctrl (Fig. 6*C*). However, all other analogs reduced apoB secretion from 40% to 80%. None of these analogs affected apoA1 secretion (Fig. 6*D*). The most potent analogs inhibiting apoB secretion were B1 and B2 (Fig. 6*C*). Both these analogs contain one copy of GalNAc at either the 5′ end or 3′ end. Introduction of multiple GalNAc-modified nucleotides, as in B5 and B8, resulted in a loss of biological activity. Thus, analogs with one GalNAc at either end are suitable for cellular delivery without liposomes.

**Table 2:** MiR-30c analogs and sequences containing GalNAc modification

Compound	Short form	Strand	Sequence (5′ to 3′)
miR30c-B1	B1	miR-30c-1-3p	(pC)UgGgAgAgGgUuGuUuAcUcC
miR30c-B2	B2	miR-30c-1-3p	CuGgGaGaGgGuUgUuUaCuC(pC)u
miR30c-B3	B3	miR-30c-1-3p	(pC)uGgGaGaGgGuUgUuUaCuC(pC)u
miR30c-B4	B4	miR-30c-2-3p	(pC)UgGgAgAaGgCuGuUuAcUcU
miR30c-B5	B5	miR-30c-1-3p	CuGgGaGaGgGuUgUuUa(pC)U(pC)(pC)u
miR30c-B6	B6	miR-30c-1-3p	(pC)UgGgAgAgGgUuGuUuA(pC)U(pC)(pC)u
miR30c-B7	B7	miR-30c-2-3p	CuGgGaGaAgG(pC)uGuUuA(pC)u(pC)U
miR30c-B8	B8	miR-30c-2-3p	(pC)UgGgAgAaGg(pC)UgUuUa(pC)U(pC)u
miR30c-B9	B9	miR-30c-1-3p	(pC)(pC)(pC)UgGgAgAgGgUuGuUuAcUcC
miR30c-B10	B10	miR-30c-2-3p	(pC)(pC)(pC)UgGgAgAaGgCuGuUuAcUcU
miR30c-B11	B11	miR-30c-2-3p	(pC)(pC)(pC)ugggagaaggcuguuuacucu

Upper case letters, 2′-deoxy-2′-fluoro (2′-F) ribosugar-modified nucleosides; lower case letters, 2′-OMe ribosugar-modified nucleosides; (pC), 2′-GalNAc-clicked cytidines.

### The GalNAc-modified miR-30c analog significantly reduces MTP activity

**Figure 7:**
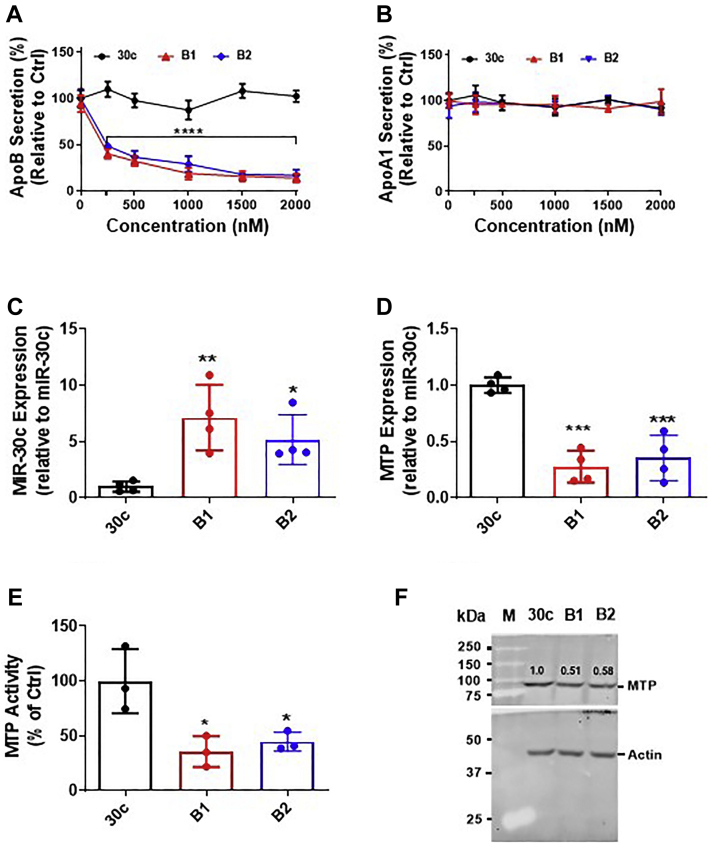
**Effects of GalNAc-modified B1 and B2 analogs on apoB secretion and MTP activity in Huh-7 human hepatoma cells.** (*A*) apoB and (*B*) apoA1 secretion (%) in the culture medium of Huh-7 cells transfected with increasing amounts of miR-30c or B1 or B2 analog without Lipofectamine RNAiMax transfection reagent. Nontransfected cells were used as controls. Data are representative of two independent experiments. *C*–*F*, cells were exposed to 250 nM of miR-30c or B1 without transfection reagent. After 48 h, the cells were used to measure (*C*) miR-30c, (*D*) MTP mRNA, (*E*) MTP activity, and (*F*) MTP levels. Data are representative of three independent experiments. Western blot analysis is from one representative experiment. After transfer, the blot was cut into two parts; the *top* was used to detect MTP, and the *bottom* was used to detect β-actin as a loading control. Changes in protein levels were quantified using ImageJ. Significance was determined at *p* < 0.05 (∗) and *p* < 0.0001 (∗∗∗∗) with two-way ANOVA (*A* and *B*), one-way ANOVA (*C*–*E*), and error bars represent mean ± SD. apoB, apolipoprotein B; miR-30c, microRNA-30c; MTP, microsomal triglyceride transfer protein.

Because analogs B1 and B2 potently inhibited apoB secretion, we performed concentration-dependent studies to assess their potency. Both analogs showed a concentration-dependent decrease in apoB secretion with an IC_50_ of 250 nM, but miR-30c had no effect (Fig. 7*A*). Under similar conditions, different concentrations of miR-30c, B1, and B2 had no effect on apoA1 secretion (Fig. 7*B*). Next, we exposed cells to 250 nM of B1 and B2 to assess the delivery of miR-30c to cells and its effects on MTP. The levels of miR-30c compared with control miR-30c transcripts were significantly higher (approximately sevenfold) in the B1-exposed cells and (approximately fivefold) in the B2-exposed cells (Fig. 7*C*). We previously showed that miR-30c diminishes apoB secretion by reducing MTP activity (23, 24, 25, 28, 29). Therefore, we studied the effects of analogs B1 and B2 on MTP expression. Both analogs B1 and B2, compared with miR-30c, reduced MTP mRNA and activity by more than 50% (Fig. 7, *D* and *E*). Western blot analysis also showed a similar decrease in MTP (Fig. 7*F*). These studies suggest that analogs B1 and B2 are likely to reduce apoB secretion in Huh-7 cells by lowering MTP expression similarly to native miR-30c.

### Modified miR-30c analogs with GalNAc and phosphorothioate at the 5′ end or 3′ end have elevated potency

**Figure 8:**
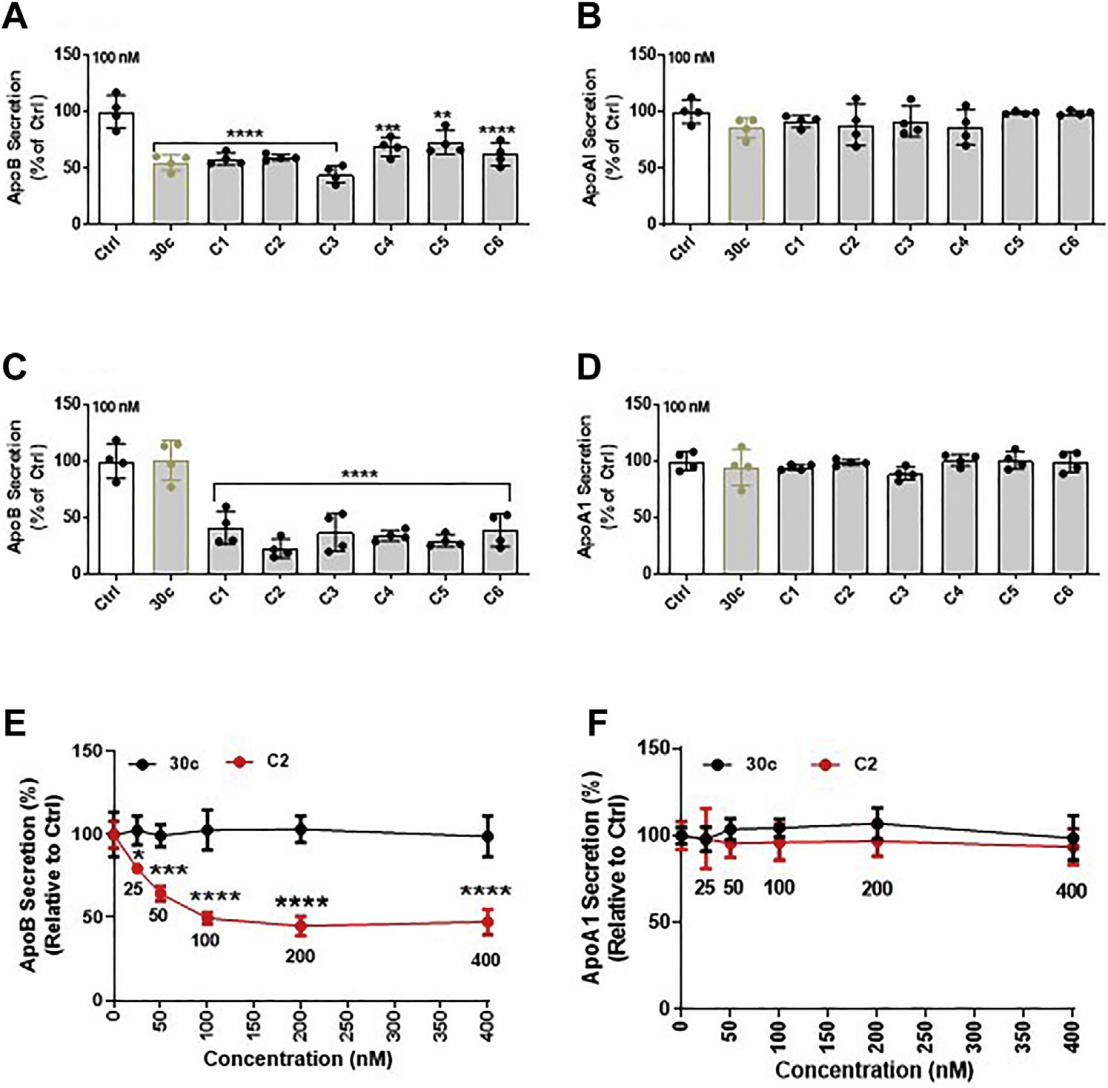
**Effects of various phosphorothioate-linked miR-30c analogs on apoB secretion in Huh-7 human hepatoma cells.** (*A*) apoB and (*B*) apoA1 secretion in Huh-7 cells transfected with Ctrl and different synthetic miR-30c series C analogs (Table 3) with Lipofectamine RNAiMax transfection reagent. MiR-30c (30c) was used as a positive control. (*C*) apoB and (*D*) apoA1 secretion in Huh-7 cells transfected with Ctrl and different miR-30c analogs without Lipofectamine RNAiMax transfection reagent. Data are representative of three independent experiments. Relative (*E*) apoB and (*F*) apoA1 secretion (%) in culture medium of Huh-7 cells transfected with increasing amounts of miR-30c or analog without Lipofectamine RNAiMax transfection reagent. Nontransfected cells were used as a control. Data are representative of two independent experiments. Significance was determined at *p* < 0.0001 (∗∗∗∗), *p* < 0.001 (∗∗∗), and *p* < 0.01 (∗∗) with two-way ANOVA (*E* and *F*), and error bars represent mean ± SD. apoA1, apolipoprotein A1; apoB, apolipoprotein B; miR-30c, microRNA-30c.

In Figure 7*A*, a significant reduction in apoB secretion was observed with 250 nM of analogs B1 and B2. Because our long-term goal is to develop analogs as potential therapeutic drugs, we focused on producing analogs that are more efficacious. Recent studies have suggested that the placement of phosphorothioate linkages improves both the specificity and silencing activity of siRNAs (37, 40). Therefore, we synthesized new series C analogs of compounds B1 and B2 by using phosphorothioate linkages (Table 3). These analogs were transfected into cells with Lipofectamine RNAiMax transfection agent and were found to inhibit apoB secretion (Fig. 8*A*) without affecting apoA1 secretion (Fig. 8*B*) in Huh-7 cells, thus suggesting that they are physiologically active. Next, we evaluated their efficacy when they were provided to cells without Lipofectamine RNAiMax transfection reagent. All analogs potently inhibited apoB secretion (Fig. 8*C*) but had no significant effect on apoA1 secretion (Fig. 8*D*). In all cases, >50% inhibition of apoB secretion was seen with 100 nM concentrations of these analogs (Fig. 8*C*). A comparison of the data in Figure 8, *A* and *C* suggested that these analogs might be more potent when delivered without lipid emulsions. Next, we verified the effects of different concentrations of the most potent analog, C2, on apoB secretion in Huh-7 cells (Fig. 8*E*). The analog C2 yielded a concentration-dependent decrease in apoB secretion, with an IC_50_ value of 20 nM. At higher concentrations, the maximum reduction in apoB secretion was ∼60% (Fig. 8*E*). At all concentrations, analog C2 had no effect on apoA1 secretion (Fig. 8*F*). These studies indicated that the addition of phosphorothioate linkages increases the efficacy of different analogs in decreasing apoB secretion and that analog C2 is a potent inhibitor of apoB secretion.

**Table 3:** MiR-30c analogs and sequences containing GalNAc and different phosphorothioate linkages

Compound	Short form	Strand	Sequence (5′ to 3′)
miR30c-C1	C1	miR-30c-1-3p	(pC)•U•gGgAgAgGgUuGuUuAcUc•C
miR30c-C2	C2	miR-30c-1-3p	(pC)•UgGgAgAgGgUuGuUuAc•U•c•C
miR30c-C3	C3	miR-30c-1-3p	(pC)•U•gGgAgAgGgUuGuUuAc•U•c•C
miR30c-C4	C4	miR-30c-1-3p	GgGaGaGgGuUgUuUaCuC(pC)•u
miR30c-C5	C5	miR-30c-1-3p	GgGaGaGgGuUgUuUaCu•C•(pC)•u
miR30c-C6	C6	miR-30c-1-3p	C•u•GgGaGaGgGuUgUuUaCuC(pC)u

Upper case letters, 2′-deoxy-2′-fluoro (2′-F) ribosugar modifications; lower case letters, 2′-OMe ribosugar modifications; (pC), 2′-GalNAc-clicked cytidine; “•” symbol represents phosphorothioate linkages.

### Effects of C2 analog on cellular lipid levels in Huh-7 human hepatoma cells

**Figure 9:**
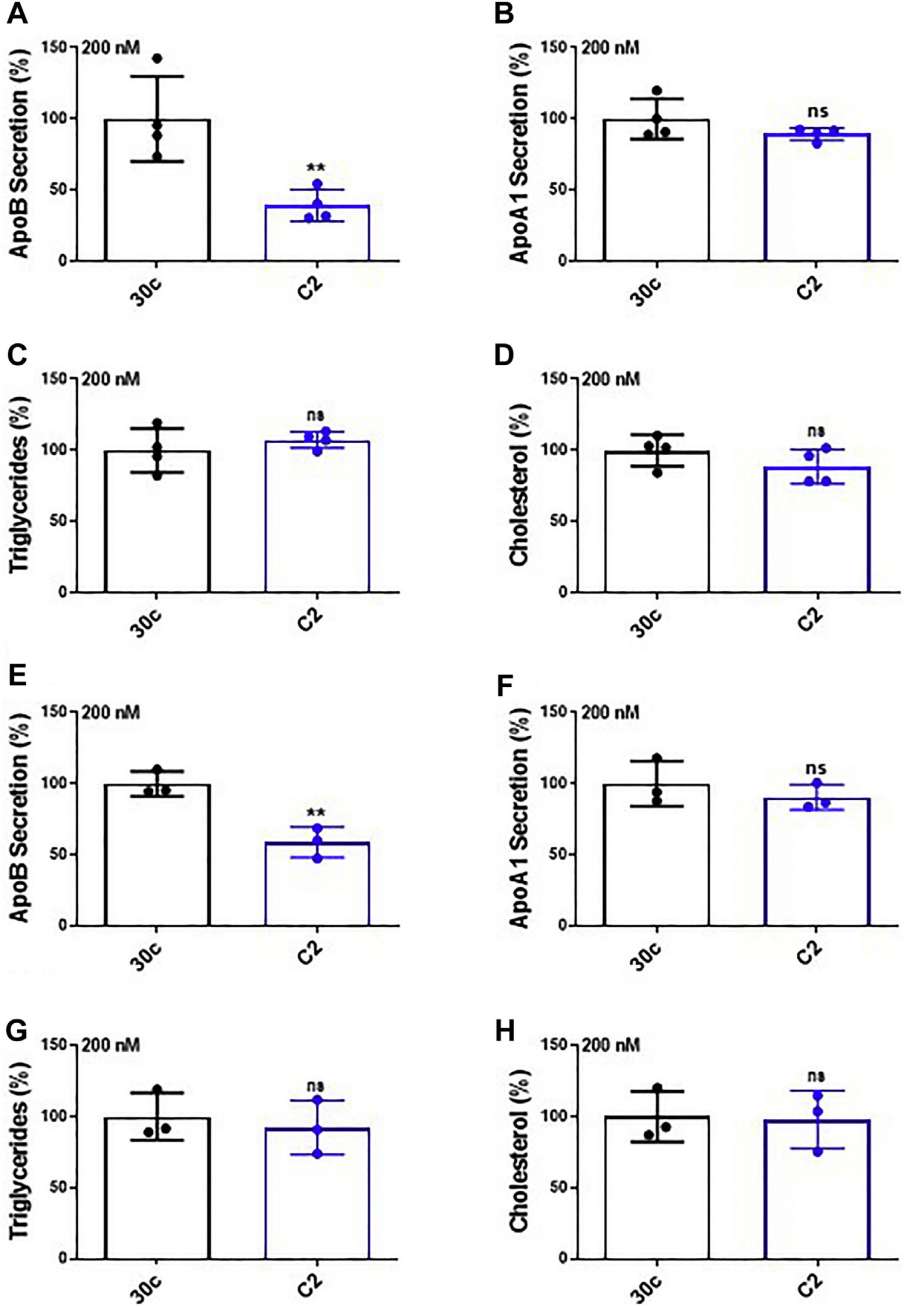
**Effects of C2 analog on lipid levels in Huh-7 human hepatoma cells and in human primary hepatocytes.***A* and *B*, Huh-7 cells were incubated in triplicate with miR-30c or C2 analog (200 nM) without Lipofectamine RNAiMax transfection reagent. After 72 h, media were changed. After 16 h, we collected media to measure (*A*) apoB and (*B*) apoA1 protein levels using ELISA. Cells were used to measure protein levels. Amounts of proteins secreted were normalized to cellular protein. Data are presented as percent of miR-30c-treated cells (control). Data are representative of two independent experiments. *C* and *D*, in separate studies, Huh-7 cells were incubated with miR-30c or analog C2 without Lipofectamine RNAiMax transfection reagent as described previously. Cells were incubated with isopropanol to extract lipids and measure (*C*) triglycerides and (*D*) cholesterol levels. Representative data of two independent studies. *E* and *F*, human primary hepatocytes were incubated in triplicate with miR-30c or C2 analog (200 nM) without Lipofectamine RNAiMax transfection reagent. Media were used to measure (*E*) apoB and (*F*) apoA1, and cells were used to measure protein levels. *G* and *H*, in a separate study, miR-30c-treated or C2 analog–treated human primary hepatocytes were used to measure cellular (*G*) triglyceride and (*H*) cholesterol levels. Significance was determined at *p* < 0.01 (∗∗) with two-tailed *t* test, and error bars represent mean ± SD (*A*–*H*). apoA1, apolipoprotein A1; apoB, apolipoprotein B; miR-30c, microRNA-30c.

It is known that reductions in apoB secretion are associated with hepatosteatosis. However, we have previously shown that miR-30c reduces apoB secretion without causing steatosis (25). Here, we investigated whether analog C2 causes steatosis or not in Huh-7 cells. As shown before (Fig. 8), analog C2 significantly reduced (>50%) apoB secretion without affecting apoA1 secretion (Fig. 9, *A* and *B*). Analog C2 had no effect on cellular triglyceride and cholesterol levels (Fig. 9, *C* and *D*). These studies showed that analog C2 reduces apoB secretion but does not increase cellular lipid levels.

### Effects of C2 analog on apoB secretion and cellular lipid levels in human primary hepatocytes

All the studies described so far were performed in Huh-7 cells. We next asked whether analog C2 also affects apoB secretion in human primary hepatocytes. Analog C2 significantly reduced apoB secretion but had no effect on apoA1 secretion in human primary hepatocytes (Fig. 9, *E* and *F*). Furthermore, reductions in apoB secretion were not associated with increases in cellular triglyceride and cholesterol levels (Fig. 9, *G* and *H*). These studies indicated that the analog C2 is a potent inhibitor of apoB secretion in hepatoma cells as well as primary hepatocytes, and this analog does not increase cellular lipid levels.

## Discussion

Our previous studies have shown that miR-30c is a good candidate to lower plasma lipids without causing hepatosteatosis. Therefore, we attempted to develop potent miR-30c analogs that could be used as potential therapeutic agents for lowering plasma lipid levels. The successful synthesis of an azido-modified GalNAc moiety (GalNAcαProN3) allowed us to selectively attach the GalNAc molecule at any location of the RNA oligonucleotide modified with an alkyne group. This synthesis design gave us the flexibility to explore the most effective modified passenger strand of miR-30c with the best cell-penetrating capacity. This design could also be extended to other miR systems. By introducing a variety of chemical modifications and phosphorothioate linkages, we improved the efficacy of the miR-30c analogs in reducing apoB secretion in Huh-7 cells. Addition of GalNAc residues at either end circumvented the need for lipid emulsions for delivery to hepatoma cells. Mechanistic studies showed that these analogs reduced MTP mRNA and thus behaved similarly to native miR-30c. Furthermore, analog C2 reduced apoB secretion in human primary hepatocytes. These studies provide evidence that the passenger strand of miR-30c can be modified to enhance cellular delivery without lipid emulsions. This system might be more cost effective than delivery with lipid emulsions.

To assess cellular toxicity, we studied apoA1 secretion, in parallel, in all studies. All the analogs had no effect on apoA1 secretion. Therefore, decreases in apoB secretion are a specific response to these analogs and were not secondary to cellular toxicity. Reductions in apoB secretion are associated with increases in cellular lipids. However, analog C2 had no effect on cellular triglyceride and cholesterol levels in Huh-7 cells and human primary hepatocytes. Therefore, these limited studies revealed no undesirable side effects.

In summary, we report the successful synthesis of various miR-30c analogs that potently inhibit MTP activity and apoB secretion. These studies corroborate our previous findings regarding the utility of miR-30c in reducing apoB secretion by liver cells and provide evidence that modification of miR-30c in the passenger strand can be useful to improve its stability and delivery into hepatoma cells. Whether these analogs can be used as therapeutic agents to lower plasma lipids and atherosclerosis in animal models remains to be determined.

## Experimental procedures

### Synthesis of chemically modified novel miR-30c-3p analogs

Anhydrous solvents were used and redistilled through standard procedures. All solid reagents were dried under a high vacuum line before use. Air-sensitive reactions were performed under argon. Analytical TLC plates precoated with silica gel F254 (Aldrich; catalog no.: 717185) were used for monitoring reactions. The ^1^H NMR spectra were measured on a Brucker Ascend 500 MHz spectrometer. Chemical shift values are reported in parts per million. High-resolution mass spectroscopy was achieved with a quadrupole time-of-flight spectrometer at the University at Albany, SUNY.

Native RNAs were custom synthesized by Integrated DNA Technologies. We used an automated RNA–DNA synthesizer (ASM800; BIOSSET Ltd) to synthesize modified antisense oligonucleotides at 1 μmol scale with commercial phosphoramidites and reagents from ChemGenes. The modified and native phosphoramidites were dissolved in anhydrous acetonitrile to obtain a concentration of 0.07 M. Trichloroacetic acid (3%) in dichloromethane was used for detritylation, and the coupling step was performed with 5-ethylthio-1H-tetrazole (0.25 M in acetonitrile) for 12 min. The unreacted 5′-OH was capped with CapA solution (80% tetrahydrofuran/10% acetic anhydride/10% 2,6-lutidine) and CapB solution (16% *N*-methyl imidazole in tetrahydrofuran). Oxidation on the phosphate backbone was performed with 20 mM iodine solution in pyridine/tetrahydrofuran/water. Phosphorothioate backbones were synthesized with sulfurization reagent (3-((dimethylamino-methylidene)amino)-3H-1,2,4-dithiazole-3-thione), instead of the iodine solution in the oxidation step. All the reagents were of oligo-synthesizer grade from ChemGenes.

The synthesis of modified miR-30c passenger strands was performed with the dimethoxytrityl-off mode of the RNA–DNA synthesizer. The protection on bases was removed, and the oligonucleotides were cleaved from the solid support with concentrated aqueous ammonium hydroxide at room temperature for 18 h. The resulting RNA solution was dried with a speed vacuum concentrator, and the pellet was redissolved with 100 μl dimethylsulfoxide and incubated with 125 μl of triethylamine trihydrofluoride (Aldrich; catalog no.: 344648) at 65 °C for 2.5 h. The RNA was precipitated by addition of 25 μl of 3 M sodium acetate solution and 1 ml ethanol and subsequent cooling of the mixture at −80 °C for 3 h before centrifugation. The RNAs were dissolved in RNase-free water and desalted again prior to storage at −20 °C in RNase-free water. The purity of modified-RNA samples was confirmed by analytical denaturing gel electrophoresis (15% polyacrylamide with 8 M urea) and electrospray ionization mass spectrometry.

### Annealing of modified miR-30c-3p strands with native miR-30c-5p strand and UV thermal denaturation studies

The modified miR-30c-3p RNAs were annealed with native miR-30c-5p in sodium phosphate buffer (10 mM, pH 6.5) containing 100 mM NaCl. The solutions were heated at 95 °C for 3 min, then cooled to room temperature at a rate of 1 °C/min, and stored overnight at 4 °C before use. For thermal denaturation studies, data points were acquired at 260 nm by heating and cooling from 5 to 85 °C (two cycles, four ramps) at a rate of 0.5 °C/min, with a Cary-300 UV–visible spectrometer equipped with a temperature controller system. The thermodynamic parameters of each duplex strand were obtained by fitting the melting curves in Meltwin software (45).

### CD spectroscopy

CD spectra were recorded at room temperature on a JASCO-815 spectropolarimeter (JASCO) over a wavelength range of 200 to 300 nm with a 1 cm path length quartz cuvette with a scanning speed of 100 nm/min, bandwidth of 1.0 nm, and digital integration time of 1.0 s. Each spectrum was averaged from four scans and baseline-corrected against the buffer.

### Synthesis of GalNAcαProN3, 3

Published protocols (46) were followed to synthesize 3-chloropropyl GalNAc 2 and GalNAcαProN3 3.

### Synthesis of 3-chloropropyl GalNAc 2

To a solution of *N-*acetyl-d-galactosamine (330 mg, 1.5 mmol) in 3-chloropropanol (5 ml), acetyl chloride (0.13 ml, 1.8 mmol) was added at 0 °C. The reaction mixture was heated at 70 °C for 15 h. The solution was concentrated, and the residue was purified by silica gel chromatography, thus yielding 3-chloropropyl GalNAc 2 (200 mg, 45%) as a white solid. TLC R_*f*_ = 0.5 (20% MeOH in CH_2_Cl_2_). ^1^H NMR (500 MHz, D_2_O) δ 4.92 (d, *J* = 4.0 Hz, 1H), 4.15 (dd, *J* = 4.4, 12.8 Hz, 1H), 4.00-3.84 (m, 4H), 3.77-3.72 (m, 4H), 3.62-3.56 (m, 1H), and 2.10-2.02 (m, 5H).

### GalNAcαProN3 3

3-Chloropropyl GalNAc 2 (200 mg, 0.671 mmol) was dissolved in CH_3_CN (6 ml) by heating the solution. NaN_3_ (436 mg, 6.71 mmol) and NaI (101 mg, 0.671 mmol) were added. The resulting mixture was stirred at 60 °C for 15 h. The solution was concentrated, and the residue was purified by silica gel chromatography, thus yielding GalNAcαProN3 3 (110 mg, 54%) as a white solid. TLC R_*f*_ = 0.4 (20% MeOH in CH_2_Cl_2_). ^1^H NMR (500 MHz, D_2_O) δ 4.94 (m, 1H), 4.21-4.18 (m, 1H), 4.03-3.94 (m, 3H), 3.85-3.78 (m, 3H), 3.59-3.47 (m, 3H), 2.08 (d, 3H), and 1.95-1.91 (m, 2H).

### Synthesis of 2′-GalNAc-modified RNA strands

The propargyl-modified RNA oligonucleotides were first synthesized according to the solid phase synthesis procedure with commercially available 2′-(O-propargyl)-phosphoramidite building blocks from ChemGenes. GalNAc-modified RNA strands were produced through mixture of the propargyl-RNA (one equivalent) with 100 equivalents of azido-modified GalNAc (GalNAcαProN3) in a 1.5 ml microcentrifuge tube. In a separate tube, 22 equivalents of copper(I) bromide (100 mM in 25% tBuOH/75% dimethylsulfoxide) and 20% acetonitrile were mixed and transferred to the RNA solution. The mixture was shaken at room temperature for 12 h. The RNA was then precipitated with 3 M sodium acetate and ethanol after storage at −80 °C for 3 h. The RNA was pelleted by centrifugation at 14,000 rpm (Eppendorf 5424) for 15 min. The RNA pellet was resuspended in 500 μl RNase-free water, and the solution was further desalted with SepPak C18 cartridges (Waters). The elution fractions with RNA were combined and concentrated with an oligonucleotide concentrator speed vacuum. The resulting click reaction products were monitored with analytical gel electrophoresis with 15% polyacrylamide containing 8 M urea.

### Cell culture studies

The Huh-7 cells were maintained in Dulbecco’s modified Eagle’s medium (DMEM) containing 10% fetal bovine serum (FBS) and 1% l-glutamine in 75 cm^2^ culture flasks with vent caps (Corning; catalog no.: 430641U) at 37 °C and 5% CO_2_ in a humidified incubator. Two types of studies were performed to assess the ability of different analogs to decrease apoB secretion. First, analogs were introduced into cells with Lipofectamine RNAiMax transfection reagent. Second, cells were exposed to different analogs without the use of any transfection reagent. In both these experiments, Huh-7 cells were seeded at a concentration of 100,000 cells/well in a 6-well plate in 2 ml of the aforementioned medium. The next day, 1 ml of fresh Opti-MEM I reduced serum medium (Gibco) (for transfection experiments) or DMEM containing 10% FBS (for nonliposome-mediated transfection) was added to the cells. The Huh-7 cells in the Opti-MEM I reduced serum medium were transfected with commercially available nonspecific control miR (Ctrl), miR-30c mimic (positive control), or our experimental novel synthetic miR-30c analogs with Lipofectamine RNAiMax transfection reagent according to the manufacturer’s protocol. Briefly, each miR was mixed with RNAiMax at a ratio of 3:1 and incubated for 30 min at room temperature. This mixture was then added to cells. In experiments testing liposome-independent delivery of miR analogs, cells in DMEM (10% FBS) were exposed to these analogs without Lipofectamine RNAiMax transfection reagent. On day 2, in both cases, 1 ml of fresh DMEM (10% FBS) was added to the cells. At 72 h after the start of transfection, the media were changed, and 1 ml of fresh DMEM (10% FBS) was added to the cells. After overnight incubation, the media were collected for apoB and apoA1 measurements. Cells were washed and collected in the presence of protease inhibitor cocktail (Sigma–Aldrich; catalog no.: P2714) for protein estimation and determination of the activity of MTP as previously described (47).

### ApoB and apoA1 measurements

The apoB levels in the collected media were determined with a human apoB ELISA development kit (MABTECH, Inc; catalog no.: 3715-1H-6) in 96-well ELISA plates (Thermo Fisher Scientific; catalog no.: 07-200-640) with 3,3′,5,5′ tetramethylbenzidine substrate (Thermo Fisher Scientific; catalog no.: 4041). The apoB concentration was calculated with apoB standard provided by the manufacturer in parallel in the same plate. The medium apoB values were normalized to the total protein in the respective wells. Total cellular protein concentrations were quantified with a Coomassie (Bradford) protein assay kit (Thermo Fisher Scientific; catalogno.: 23200). The apoB concentrations in the media of control miR (Ctrl)-transfected cells were set at 100%. ApoB secretion by cells exposed to miR-30c mimic or newly synthesized miR-30c analogs is presented as a percentage of this value.

The apoA1 levels in the collected media were determined with a human apolipoprotein A-I/ApoA1 DuoSet ELISA kit (R&D Systems; catalog no.: DY3664) in 96-well ELISA plates (Thermo Fisher Scientific; catalog no.: 7-200-640). Substrate (catalog no.: DY999) and stop (catalog no.: DY994) solutions were from R&D Systems. The apoA1 concentration was calculated with an apoA1 standard curve prepared in parallel with standards provided by the manufacturer (R&D Systems; catalog no.: DY3664). ApoA1 levels in the media were normalized to total protein in the respective wells. The concentrations of apoA1 in the control miR (Ctrl)-transfected cells were set to 100%, and those in miR-30c mimic–treated cells were normalized to this value.

### Measurement of miR-30c and MTP transcript levels

For miR-30c quantification by quantitative RT–PCR, complementary DNA (cDNA) was synthesized from RNA isolated from cells with a TaqMan MicroRNA Reverse Transcription kit (Applied Biosystems; catalog no.: 4366597). The miR-30c and U6-specific primers were purchased from Thermo Fisher Scientific. For quantitative RT–PCR, TaqMan Universal Master Mix II (Applied Biosystems; catalog no.: 4440043) was used. For miR-30c quantification, the Ct method with normalization to U6 was used, and the data are presented as fold changes.

For MTP quantification by quantitative RT–PCR, cDNA was synthesized from isolated RNA with a High-Capacity cDNA Reverse Transcription Kit (Thermo Fisher Scientific; catalog no.: 4368813). The human MTP and β-actin-specific primers were purchased from Integrated DNA Technologies (MTP primers, 5′-TGTGGCCTTACTATGGAGGAA-3′ and 5′-AAGGAGCGTAGGTCTTTGCAG-3′; β-actin primers, 5′-AGAGCTACGAGCTGCCTGAC-3′ and 5′-AGCACTGTGTTGGCGTACAG-3′). For quantitative RT–PCR, PowerTrack SYBR Green Master Mix (Thermo Fisher Scientific; catalog no.: A46109) was used. For MTP quantification, the Ct method with normalization to β-actin was used, and data are presented as fold change with respect to controls.

### MTP levels and activity measurements

Transfected Huh-7 cells were washed with ice-cold PBS and scraped from the wells in ice-cold buffer K (1 mM Tris–HCl, 1 mM EGTA, and 1 mM MgCl_2_, pH 7.6) containing protease inhibitor cocktail (Sigma–Aldrich; catalog no.: P2714). Cells were manually lysed through 20 passes through a BD PrecisionGlide 25G needle, and the total protein concentrations were measured with a Coomassie (Bradford) protein assay kit (Thermo Fisher Scientific; catalog no.: 23200). Proteins (25 μg) were resolved by SDS-PAGE (10%). A polyclonal rabbit primary antibody to human MTP (Abcam; catalog no.: ab63467) and a monoclonal rabbit antibody to β-actin (Cell Signaling Technology; catalog no.: 8457) were used at 1:1000 dilution. Anti-rabbit immunogobulin G, horseradish peroxidase–linked secondary antibody (Cell Signaling Technology; catalog no.: 7074) was used at 1:2000 dilution. The blots were developed with a ChemiDoc Touch Imaging system (Bio-Rad). To determine the MTP activity, 50 μg of total proteins was used. Fluorescently labeled triglyceride transfer assays were performed as previously described (25, 28, 29, 47).

### Effects of C2 analog on apoB secretion and lipid levels in human primary hepatocytes

To extend our studies about the efficacy of different miR-30c analogs beyond human hepatoma Huh-7 cells, we purchased human primary hepatocytes (H1000.H15B+; lot no.: HC4-25) from Sekisui XenoTech. Cells were thawed using Sekisui XenoTech’s thawing protocol and OptiThaw Hepatocyte Kit. After seeding in collagen-coated plates, cells were treated with the analog C2. After 72 h, media were collected to measure apoB and apoA1 levels using ELISA. Cells were collected in 0.1 N NaOH to measure protein levels. Secretion of apoB and apoA1 was normalized to cellular protein. Data are presented as control of miR-30c.

To determine whether analog C2 increases cellular lipid levels, cells were washed with PBS. To each well, 500 μl of isopropanol was added, and plate was incubated overnight at 4 °C. Next day, we collected supernatants, dried, and resuspended in 100 μl of isopropanol to measure total triglycerides and cholesterol using commercial kits (Pointe Scientific). To each well, 500 μl of 0.1 N NaOH was added to determine the protein concentration for normalization. Lipid levels were normalized to protein levels, and data from miR-30c-treated cells were used as control.

### Statistical analysis

We performed statistical analysis in GraphPad Prism, versions 8 and 9 (GraphPad Software, Inc). Significance was determined with *t* test (two-tailed), one-way ANOVA, and two-way ANOVA. All data are represented as mean ± SD. The symbols ∗, ∗∗, ∗∗∗, and ∗∗∗∗ represent significance at *p* < 0.05, *p* < 0.01, *p* < 0.001, and *p* < 0.0001, respectively.

## Data availability

All the data are included in the article.

## Conflict of interest

The authors declare that they have no conflicts of interest with the contents of this article.
